# The pathogenesis of macrophage activation syndrome

**DOI:** 10.1186/1546-0096-12-S1-I7

**Published:** 2014-09-17

**Authors:** Fabrizio De Benedetti

**Affiliations:** 1 Ospedale Bambino Gesu, Roma, Italy

## 

The term macrophage activation syndrome (MAS) identifies a severe and potentially fatal complication of s-JIA, and, more rarely of other rheumatic diseases. MAS share similarities in clinical features and laboratory abnormalities with primary and secondary heamophagocytic lymphohystiocytosis (HLH). Indeed it is currently classified among secondary HLH and the term rheuma-HLH has been used to indicate this condition. The clinical and laboratory similarities with primary genetic- caused HLH led to the hypothesis that pathogenic mechanisms leading to the typical features of MAS/rheuma-HLH are similar to those involved in primary HLH. We will review the evidence supporting this hypothesis; particularly the role of hyper-responses to TLR activation, of subclinical variants of genes involved in the cytotoxic pathways, and of the transient NK cytotoxicity defect induced by inflammatory cytokines. We will also present evidence on the role of IL-6, IL-1 and IFN-g in this syndrome and discuss the potential benefits of therapies targeted to these cytokines.

## Disclosure of interest

F. De Benedetti Grant / Research Support from: Sobi, Novimmune, Novartis, Roche, Pfizer, Abbvie

